# Acknowledgement to Reviewers 2020

**DOI:** 10.1259/bjrcr.20219001

**Published:** 2021-03-12

The Editors gratefully acknowledge the contribution of the following colleagues who acted as reviewers for *BJR*|*Case reports* in 2020.

Mohamed Abd Ellah

Sharif Abdullah

Shoko Abe

Mohamed Abou El-Ghar

Giancarlo Addonisio

Himanshu Agarwal

Abhinav Aggarwal

Anjali Agrawal

Archi Agrawal

Saima Ahmad

Zahoor Ahmed

Mohamed Nadeem Ahmed

Domenico Albano

Rosemary Allan

Robert Allison

Ali Alsafi

Michele Altiero

Maria Chiara Ambrosetti

Roberta Ambrosini

Constantinos Anagnostopoulos

Jayajothi Anandapadmanabhan

Kirsty Anderson

Sarit Appel

Ankur Arora

Abhishek Arora

Supreeta Arya

Monika Arzanauskaite

Julian Atchley

Swapndeep Atwal

Daly Avendano

Giacomo Avesani

Warren Bacorro

Joaquim Barreto Oliveira

Biradar Basavaraj

Raffaella Basilico

Davide Bellini

Michele Bertolotto

Venkatraman Bhat

Lorenzo Biassoni

Charles Bishop

Christian Blüthgen

Matteo Bonatti

Enrico Boninsegna

Rajesh Botchu

William Boteler

Costanza Bruno

Maria Ludovica Carerj

Aldo Carnevale

Orlando Catalano

Francesca Catanzariti

Antonino Cattafi

Marco Cavallaro

Leslie Chacko

Anthony Chambers

Priyank Chatra

Udit Chauhan

Ashish Chawla

Mohd Firdaus Che Ani

Chung Shen Chean

Sam Chippington

Li Shyan Ch'ng

Anchalee Churojana

Demosthenes Cokkinos

Manuel Conson

Alberto Contro

Peter Corr

Renzo Corvò

Clemens Cyran

Giancarlo d’Andrea

Çağrı Damar

Karthikeyan Damodharan

Alessandro D'Amuri

Roberta Danzi

Indra Das

Tilak Das

Pim de Graaf

Manu Dhillon

Alper Dilli

Cedric Draulans

Juliana Duarte

Karen Duncan

Mainak Dutta

Mahendra Dwiwedi

Mat Elameer

Mohammad Elsayed

Joanna Fairhurst

Maria Cristina Firetto

Bernie Foran

Giovanni Foti

Matthew Gibson

Samuel Gitau

Sofia Gourtsoyianni

Ruth Green

Jochen Grimm

Shabnam Grover

Susanna Guerrini

Abhishek Gulia

Windsor Gunawardena

Saumya Gupta

Kovacs Gyoergy

Nigel Hoggard

John Holemans

John Howells

Isabella Iadevito

Klaus Irion

Aniket Jadhav

Rohan Jagtap

Neeraj Jain

Judy James

Ehsan Jangholi

Eduardo Jayme

Sanjay Jeganathan

Devimeenal Jegannathan

Musa Kaleem

Thomas Kane

Ravindran Karthigan

Irfan Kayani

Sairah Khan

Rohit Khandelwal

Amithavikrama Kheda

David Kim

Michail Klontzas

Zehra Pinar Koc

Ravi Kothari

Tom Kovitwanichkanont

Wilhelm Kuker

Saminderjit Kular

Atin Kumar

Akira Kunimatsu

Francesco lassandro

Federica Leone

Cindy Leung

Steven Lev

Francesco Maria Lo Russo

Fabio Lombardo

Elaine Lui

Matthew Lukies

Shashikant Mane

Rishi Mathew

Maria Antonietta Mazzei

Caroline McCann

Mohamad Syafeeq Faeez Md Noh

Hakim Megadmi

Iosif Mendichovszky

Katsuhiro Mizutani

Homan Mohammadi

Hilary Moss

Jader Muller

Sankar Neelakantan

Luca Nicosia

Selim Nural

Rosa Elena Ochoa Albíztegui

Curtis Offiah

Marcello Orsi

Emre Pakdemirli

Kavitha Palled

Om Biju Panta

Dimitri Parra

Flavius Parvulescu

Vassiliki Pasoglou

Aruna Patil

John Pattison

Wilfred Peh

Deborah Pencharz

Alessandra Perillo

Federica Perillo

Kostas Perisinakis

Jane Phillips-Hughes

Roberto Picascia

Stefano Giusto Picchi

Isabella Plumptre

John Port

Sunil Puri

Neeraj Purohit

Syed Qaseem

Mark Radon

Ashok Raghavan

Hamid Rajebi

Sameer Raniga

Anuradha Rao

Abdel Razek

Denis Remedios

Luigia Romano

Federica Romano

Maxime Ronot

Nicholas Rowell

Giovanni Rusconi

Zia Saad

Anju Sahdev

Rakesh Sajjan

Prashant Sankaye

Radha Sarawagi

Deepali Saxena

Rüdiger Schernthaner

Sana Shaikh

Manjunath Shekar

Sumer Shikhare

Shivakumar Swamy Shivalingappa

Philip Shorvon

Giacomo Sica

Salvatore Silipigni

Dimpi Sinha

Carmelo Sofia

Aikaterini Solomou

Vijay Somasundaram

Alessandro Stecco

Konstantinos Stefanidis

David Stobo

Chun Qiu Su

Kia Sing Tan

Yen Zhi Tang

Niluja Thiruthaneeswaran

Christine Tolman

Michele Tonerini

Yutaka Toya

Virginia Tsapaki

Nikolaos Tselis

Yusuke Uchiyama

Manuela Vadrucci

Stefano Vagge

Anja van der Kolk

Hanneke van Santen

Sobhan Vinjamuri

Andre Tjie Wijaya

Cheng Xie

